# A Multiscale Evaluation of Erbium-Doped Yttrium-Aluminum-Garnet Laser Osteotomy: Integrating Macroscopic and Cellular Analyses

**DOI:** 10.3390/bioengineering13020237

**Published:** 2026-02-18

**Authors:** Anjie Shen, Boxuan Huang, Hang Bao, Teng Zhang, Kaijun Zhang, Bin Zhao, Haoyuan Du, Junqiang Wang, Wei Han

**Affiliations:** 1 Department of Orthopedic Trauma, Peking University Fourth School of Clinical Medicine, Peking University, Beijing 100035, China; 2311110589@stu.pku.edu.cn (A.S.);; 2 Department of Orthopedic Trauma, Beijing Jishuitan Hospital, Capital Medical University, Beijing 100035, China; 3 National Center for Orthopaedics, Beijing 100035, China

**Keywords:** laser bone surgery, orthopedic surgery, precision osteotomy, bone healing, non-contact ablation

## Abstract

Background: Traditional high-speed mechanical osteotomes cause substantial thermal and mechanical trauma, impairing bone healing. Erbium-doped yttrium-aluminum-garnet (Er:YAG) lasers, with water-mediated non-contact ablation, offer precise osteotomy potential with minimal collateral damage. This study demonstrated the feasibility of Er:YAG laser use for complex osteotomies and elucidated its multi-scale biological impacts on bone. Methods: A custom Er:YAG laser performed Z/arc-shaped osteotomies on fresh ovine bone (oscillating saw as control); paired rat tibial osteotomies; and compared laser vs. saw resection. Osteotomy surfaces were characterized by SEM/micro-CT; histological staining quantified thermal/mechanical damage. Bone marrow-derived mesenchymal stem cell (BMSC) adhesion, viability, and infiltration on cut surfaces were evaluated via LSCM. Result: In the ex vivo ovine model, the Er:YAG laser enabled precise execution of complex osteotomies (Z-shaped and arc-shaped), producing significantly narrower gaps than the oscillating saw (1.14 mm vs. 2.70 mm, *p* < 0.001) with high geometric fidelity and smooth surfaces free of burrs, micro-cracks, or debris. In the in vivo rat model, laser ablation simultaneously minimized both thermal and mechanical damage at the osteotomy interface: it reduced the thermal damage depth (154 vs. 592 µm, *p* < 0.001) and empty lacunae rate (16.8% vs. 41.8%, *p* < 0.001) while completely avoiding the mechanical damage zone (297 µm) induced by sawing. Furthermore, the laser-ablated surface established a highly bioactive interface, which significantly enhanced the adhesion (606 vs. 389 cells), viability (86.9% vs. 46.6%), and infiltration depth (196 vs. 75 µm) of bone marrow-derived mesenchymal stem cells (all *p* < 0.001). Conclusions: In conclusion, this proof-of-concept study demonstrates that the Er:YAG laser has the potential to enable precise bone resection while preserving microstructure. By establishing a pro-regenerative microenvironment, this technology shows promise as a biologically favorable alternative to conventional sawing, although further technical refinement and long-term validation are essential for its clinical translation.

## 1. Introduction

Millions of patients worldwide undergo orthopedic surgeries annually [1,2], in which precise osteotomy—the surgical cutting of bone—is a fundamental procedure. Currently, the clinical standard relies on high-speed mechanical tools such as metal drills and oscillating saws. While straightforward, this approach carries inherent drawbacks: the frictional heat generated during sawing can cause substantial thermal damage to osteocytes [3]; the resulting bone debris not only obstructs the surgical field but may also induce aseptic necrosis [4] and, by prolonging macrophage-mediated debridement, delay bone regeneration [5]. Moreover, operating on morphologically complex bony regions (e.g., pelvis, spine) poses significant technical challenges even for skilled surgeons [6,7].

To minimize iatrogenic trauma associated with high-speed instruments, piezoelectric devices (piezosurgery) have been increasingly utilized in specialized orthopedic and dental osteotomies. These instruments deliver high precision and lower the risk of thermal necrosis [8]; yet they remain contact-dependent cutting tools. Their clinical application is often limited by poor cutting efficiency in dense cortical bone—especially in diaphyseal regions—whereby operative time can be considerably prolonged. A further persistent drawback is the risk of tip fracture under high-resistance conditions, which may undermine surgical safety and disrupt procedural continuity [9,10]. Consequently, there remains an ongoing demand for non-contact osteotomy technologies that balance high geometric precision and biological safety with the capacity to optimize operative performance. Lasers, since their inception, have found successful applications in ophthalmology, dermatology, and dentistry [11,12]. Early skepticism regarding laser bone ablation stemmed from observations of severe surface carbonization and impaired healing induced by certain laser types (e.g., CO_2_ lasers) [13]. However, a breakthrough has emerged with the erbium-doped yttrium aluminum garnet (Er:YAG) laser, owing to its specific wavelength. The Er:YAG laser emits at 2940 nm, which is intensely absorbed by water in bone tissue. This property theoretically enables efficient ablation while restricting thermal diffusion [14]. Preliminary studies have demonstrated the feasibility and safety of Er:YAG laser osteotomy in small animal and ex vivo models [15,16], and some evidence suggests it may enhance bone healing [17,18]. Nevertheless, a systematic and mechanistic explanation for these observations is still lacking.

Therefore, this study aimed to comprehensively evaluate the potential of Er:YAG laser for complex osteotomy using a custom-built laser platform. We first verified its macro-scale feasibility for executing complex geometric cuts in a large ex vivo bone model [19,20]. Subsequently, in a small animal model, we conducted a multi-scale analysis—encompassing morphological, histological, and cytobiological assessments [21,22]—to thoroughly elucidate the morphological characteristics and biological effects of bone following laser ablation. This work seeks to provide foundational data to support the potential clinical translation of this technology.

## 2. Materials and Methods

### 2.1. Laser System

**Figure 1:**
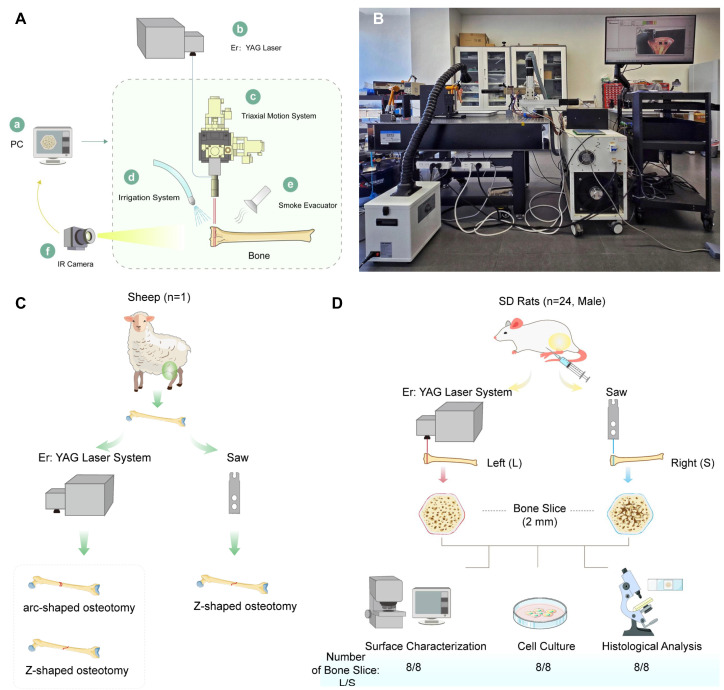
Schematic of the experimental setup and animal study design. (**A**) Schematic diagram of the custom-built Er:YAG laser osteotomy platform. Key components: (a) PC control unit, (b) solid-state Er:YAG laser source, (c) three-dimensional motion system (equipped with longitudinal and transverse motors and a focusing device), (d) integrated spray cooling device, (e) debris removal suction device, and (f) real-time monitoring camera. (**B**) Photograph of the actual experimental platform corresponding to the schematic in (**A**). (**C**) Schematic of ex vivo complex osteotomy procedures on fresh ovine bone specimens. Z-shaped and arc-shaped osteotomies were performed to evaluate macroscale feasibility and precision. (**D**) Schematic of the in vivo paired-design study in rats. Bilateral tibial osteotomies were conducted, with one side subjected to laser ablation (Group L) and the contralateral side to conventional oscillating saw (Group S) as self-control.

Laser ablation of bone tissue was performed using a pulsed Erbium-doped Yttrium Aluminum Garnet (Er:YAG) laser system, as shown in Figure 1A,B. The laser was mounted on a three-axis motion platform to enable osteotomy of complex-shaped bone structures. A 200 mm focal length lens was employed to generate a 1 mm diameter laser spot at the target bone surface. This dimension was selected to align with the 1 mm thickness of standard oscillating saw blades, which are widely utilized in conventional orthopedic osteotomy. To protect the optical assembly, a protective cover was installed over the focusing unit to prevent contamination or damage from ejected bone debris. To manage thermal effects, a concurrent water-cooling system was directed at the osteotomy site to dissipate heat and minimize thermal accumulation in the bone tissue during and after ablation. Simultaneously, a suction system was employed at the ablation site to continuously remove generated bone debris, thereby minimizing potential shielding effects and maintaining consistent ablation efficiency.

The laser parameters adopted in this study were as follows: single-pulse energy 1.2 J, pulse width 400 µs, frequency 10 Hz, scanning speed 10 mm/s, corresponding to an energy density of 1.528 J/mm^2^. Normal saline was used for cooling during the scanning process. The laser parameter settings are presented in Table 1.

### 2.2. Animals

Fresh ovine bone specimens were obtained from a 1-year-old donor (body weight approximately 50 kg) from a local abattoir. The time interval between animal euthanasia and experimental processing was restricted to 4 h. For subsequent histological and cytological analyses, 24 male Sprague-Dawley (SD) rats (10 weeks old) were purchased from Beijing Vital River Laboratory Animal Technology Co., Ltd. (Beijing, China). All rats were acclimated for at least 1 week before experimentation under standard laboratory conditions (12 h light/dark cycle, 22 ± 2 °C, 50 ± 10% relative humidity), with free access to food and water.

### 2.3. Experimental Setup

#### 2.3.1. Complex Osteotomy on Ovine Bone

Complex osteotomies, including Z-shaped and arc-shaped configurations, were performed on cortical bone segments harvested from the diaphyses of freshly isolated ovine femurs and tibias using the aforementioned laser system. For comparison, Z-shaped osteotomies were conducted on similar specimens using a standard orthopedic oscillating saw (BoJin Medical Co., Ltd., Shanghai, China) equipped with a 1 mm thick blade from the same manufacturer (see Figure 1C).

#### 2.3.2. Rat Bone Comparative Study and Sample Allocation

Twenty-four 10-week-old male SD rats were euthanized following intraperitoneal anesthesia with pentobarbital sodium, and bilateral tibiae were harvested. A paired experimental design was adopted: the left tibia from each rat served as the experimental group (Group L) and underwent laser osteotomy, while the contralateral right tibia served as the internal control group (Group S) and underwent mechanical saw osteotomy. To ensure intra-operative consistency, all mechanical osteotomies were performed by a single senior orthopedic surgeon using a standard oscillating saw operated at 18,000 cycles/min. A standardized manual pressure of approximately 5 N was maintained to simulate the tactile feedback of clinical bone resection. To maintain consistent cutting efficiency and minimize potential confounding thermal effects from blade wear, the oscillating saw blade was replaced every four rats, with a total of six standardized blades used for the 24 rats in the study. Throughout the procedure, the cutting site was continuously irrigated with room-temperature saline at a flow rate of 10 mL/min. This cooling protocol was designed to synchronize with that of the laser group, thereby mitigating thermal confounding variables. Transverse osteotomies were performed at the proximal metaphysis of all tibiae, at a level 5 mm distal to the tibial articular surface, yielding 24 pairs of proximal tibial bone blocks. These blocks were then allocated into three analytical cohorts: eight pairs for surface morphological observation, eight pairs for histological analysis, and the remaining eight pairs for cytological behavior evaluation (see Figure 1D).

### 2.4. Surface Topography and Geometric Accuracy Analysis

The surface topography and geometric accuracy of the ovine bone osteotomies were quantitatively assessed. To evaluate the micro-scale surface quality, bone specimens were prepared into planar blocks. The surface topography of the cut surface was characterized using a confocal laser scanning microscope (LSM 900, Zeiss, Oberkochen, Germany). For each specimen, a representative 2.5 mm × 2.5 mm region was randomly selected on the osteotomy surface. The system reconstructed the three-dimensional (3D) morphology by scanning and identifying the peak focus height for each pixel at a lateral resolution of 2.5 μm. The 3D surface roughness, specifically the root-mean-square height (S_q_), was subsequently calculated using the integrated post-processing software.

For macro-geometric evaluation, the width was measured at five pre-defined, equidistant points along each side of the cut using a digital caliper (Mitutoyo, Kanagawa, Japan). The average of these measurements was calculated and reported as the mean gap width for that specimen. For the Z-shaped osteotomies, the two internal angles were measured using a digital angle finder. For the arc-shaped osteotomy, the central arc angle was determined.

### 2.5. Surface Morphology Analysis

Freshly excised bone blocks were gently rinsed with phosphate-buffered saline (PBS) to remove any residual bone debris, blood cells, and bone marrow tissue. Subsequent analyses were performed as follows:

#### 2.5.1. Observation Under Light Microscopy

Bone samples were evaluated for cross-sectional morphology using a 10× objective on a light microscope (DSX1000, Olympus, Tokyo, Japan).

#### 2.5.2. Scanning Electron Microscopy (SEM)

Bone specimens were initially fixed in 2.5% glutaraldehyde, followed by sequential dehydration through a graded ethanol series and critical point drying. To ensure surface conductivity, the samples were sputter-coated with a 5 nm gold (Au) layer. The ultrastructural surface morphology was then examined using a field-emission scanning electron microscope (Zeiss Gemini 300, Carl-Zeiss, Oberkochen, Germany). Imaging was conducted at an accelerating voltage of 15 kV and a working distance of 10 mm, ensuring high-resolution capture of the laser-induced topographical changes.

#### 2.5.3. Laser Scanning Confocal Microscopy (LSCM)

The bone blocks were placed in confocal dishes and imaged using a laser scanning confocal microscope (LSCM; LSP900, Carl-Zeiss, Germany). The scanning area was set to 1400 μm × 1400 μm × 600 μm, with a step size of 1 μm. The three-dimensional structure of trabeculae at the cut surface was reconstructed using ZEN blue edition software (Version 3.38, ZEISS, Oberkochen, Germany), providing high-resolution images along the z-axis.

#### 2.5.4. Micro-CT Analysis

Samples were scanned using a micro-CT system (Skyscan, Bruker, Kontich, Belgium). To quantify the immediate structural integrity and debris accumulation at the interface, a cylindrical volume of interest (VOI) was defined. For the experimental groups, this VOI was established using the osteotomy surface (located 5 mm distal to the proximal articular surface of the tibia) as the reference plane, extending 200 μm proximally into the proximal bone fragment. The radius was set at 3 mm to encompass the entire cortical and trabecular area. For the blank control group, the VOI was positioned at an identical anatomical location, precisely 5 mm distal to the proximal articular surface, to ensure a valid baseline comparison. Quantitative analysis was performed using CTAn software (v.1.18, Bruker, Belgium). To ensure an unbiased comparison of bone volume/total volume (BV/TV) and trabecular thickness (Tb.Th) across all groups, a global thresholding strategy (consistently set at 60–80 grayscale units) was applied to segment calcified tissue from the background. This threshold range was determined based on visual inspection of the grayscale histograms to optimize the detection of both intact trabeculae and iatrogenic debris. Three-dimensional reconstruction and subsequent quantitative analysis were performed accordingly.

### 2.6. Histological Analysis

Samples were decalcified in 10% ethylenediamine tetraacetic acid (EDTA) for 3 weeks, embedded in paraffin, and sectioned perpendicular to the osteotomy surface into 5-μm slices. The sections were stained with hematoxylin and eosin (H&E) for histological evaluation. Bright-field images were captured using an inverted fluorescence microscope (Pannoramic 250FLASH, 3DHISTECH, Budapest, Hungary). To quantify osteotomy-induced damage, the depths of the mechanical and thermal injury zones were measured using ImageJ software (version 1.54p, National Institutes of Health, Bethesda, MD, USA).

The histological sections were evaluated to quantify iatrogenic injuries based on predefined, objective criteria. Thermal damage was defined by the presence of: (1) empty bone lacunae, indicating the loss of osteocyte nuclei; (2) pyknotic nuclei, characterized by shrunken and hyperchromatic chromatin; (3) hypereosinophilic condensation of the bone matrix; and (4) clusters of necrotic cells at the osteotomy margin. The depth of thermal damage was measured from the cut surface to the most distal point exhibiting these features. Furthermore, the empty lacunae rate was quantified to assess cellular viability within a 200 um-wide region extending from the osteotomy margin. This rate was defined as the percentage of empty lacunae relative to the total number of lacunae (the sum of nucleated and empty lacunae) identified within this standardized zone. [23,24,25]. Mechanical damage was defined by: (1) obvious fragmentation of the bone matrix; (2) structural distortion of the vascular canals (formerly termed Haversian systems in large animals) or trabecular architecture; and (3) the accumulation of compacted bone debris. The mechanical damage zone was quantified as the thickness of the disrupted tissue layer immediately adjacent to the interface [26].

### 2.7. Biological Effects Analysis

#### 2.7.1. Reagents and Cell Culture

Bone marrow-derived mesenchymal stem cells (BMSCs, CAS: CP-R131) and specialized medium (CAS: CM-R131) were procured from Procell Company (Wuhan, China). Cells were cultured in complete cell culture medium at 37 °C with 5% CO_2_. Cell density reached 80–90%, cells were passed to the next generation at a ratio of 1:3. The cell density of all cell experiments was 8 × 10^4^ cells/well.

#### 2.7.2. Live/Dead Cell Staining

Bone blocks were disinfected by immersion in a triple-antibody solution (Solarbio, Beijing, China) for 1 h and placed in a 24-well plate, with each well supplemented with 500 μL of specialized medium to fully immerse the blocks. A predetermined number of BMSCs were then seeded onto each bone block and gently resuspended for uniform distribution, followed by incubation at 37 °C and 5% CO_2_ for 72 h. Subsequently, the medium was removed, bone blocks were rinsed with sterile PBS, and live/dead staining was performed using a Calcein/PI Viability Assay Kit (Beyotime, Shanghai, China) under light-protected conditions at 37 °C for 30 min. Finally, stained blocks were imaged via LSCM under a 10× objective with layer scanning at 494 nm and 617 nm wavelengths (200 μm depth), and Image J software was used to quantify live/dead cells, calculate viability, and assess cellular penetration depth into the bone blocks.

#### 2.7.3. Cytoskeleton Staining and Adhesion Analysis

Bone blocks were prepared as described above. Subsequently, BMSC cells were seeded onto the bone surfaces at 37 °C for 48 h, rinsed with PBS, and fixed with 4% paraformaldehyde (PFA) at room temperature for 10 min. The cytoskeleton was stained with phalloidin (Beyotime, China) for 15 min at room temperature, and the nuclei were stained with 4′,6-diamidino-2-phenylindole (DAPI) (Beyotime, China) for 15 min, protected from light. Fluorescent signals were examined using an LSCM (LSP900, Carl-Zeiss, Oberkochen, Germany).

### 2.8. Statistical Analysis

Statistical analysis was performed using SPSS software (version 25.0, IBM Corp., Armonk, NY, USA). For continuous data from the ovine model (e.g., osteotomy gap width), the Shapiro–Wilk test was used to assess normality, and Levene’s test to verify homogeneity of variances. When the data met these assumptions, an independent-sample *t*-test was applied for comparisons between the laser and saw groups. Continuous data from the rat model were confirmed to follow a normal distribution via the Kolmogorov–Smirnov test, and all continuous data are presented as mean ± standard deviation. For paired data from the rat model (bilateral tibial osteotomy), a paired-sample *t*-test was used for intragroup comparisons. A *p*-value < 0.05 was considered statistically significant. To minimize observer bias, all subsequent quantitative measurements were performed independently by two investigators blinded to the experimental grouping through the use of randomized numerical codes. The mean values were reported, and inter-observer reliability was evaluated using the intraclass correlation coefficient (ICC).

### 2.9. Ethical Statement

This study and the included experimental procedures were approved by the Biomedical Ethics Committee at Beijing Jishuitan Hospital, Capital Medical University (approval number: jldszd-2025-02-03, approval date: 14 February 2025). And all experimental methods were performed in accordance with relevant guidelines and regulations, including the ARRIVE guidelines and Regulations for the Administration of Affairs Concerning Experimental Animals of P. R. China.

## 3. Results

### 3.1. Measurement Reliability

The intraclass correlation coefficient was between 0.83 and 0.93 for inter-observer reliability. These results confirm the high consistency and reproducibility of the measurement protocols used in this study (Supplementary Table S1).

### 3.2. Performing Laser Osteotomy of Ovine Bones

**Figure 2:**
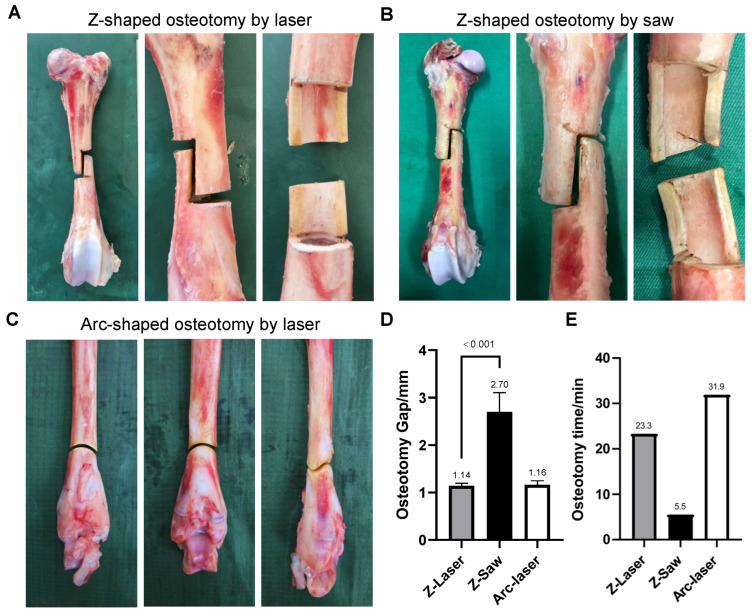
Macroscopic precision of laser osteotomy for ovine bones. (**A**) Z-shaped osteotomy performed with the Er:YAG laser on an ovine femur specimen. It can be seen that the osteotomy surface was smooth and without carbonization. (**B**) Z-shaped osteotomy performed with an oscillating saw on an ovine femur specimen. The surface of the osteotomy is rough, with obvious signs of carbonization, and iatrogenic fractures can be seen. (**C**) Arc-shaped osteotomy achieved using the Er:YAG laser on an ovine tibia specimen. (**D**) Quantitative comparison of osteotomy gap width between laser and saw for the Z-shaped osteotomy. (**E**) Comparison of the duration of different osteotomies.

As shown in Figure 2 and Table 2, the ovine bone samples were successfully osteotomized into both Z-shaped and arc-shaped sections using the laser system. For the Z-shaped section, the two angles were 93° and 88°, respectively, the resulting osteotomy gap measured 1.14 ± 0.05 mm, and the operation time was 23 min 15 s. For the arc-shaped osteotomy, the arc angle was 125°, the gap was 1.16 ± 0.08 mm, and the operation time was 31 min 47 s. Multi-planar observation (sagittal, coronal, transverse) confirmed that all laser-osteotomy surfaces were smooth, devoid of burrs, micro-cracks, or debris. Quantitative surface topography analysis further corroborated these findings, with the laser-processed surfaces exhibiting a low 3D surface roughness (S_q_) of 15.79 μm.

In contrast, the osteotomy performed with the oscillating saw yielded a wider, irregular, and poorly controlled gap. Quantitative measurements revealed an average gap width of 2.70 ± 0.41 mm, with individual values ranging from 2.2 to 3.3 mm, representing a significantly wider osteotomy compared to the laser group (*p* < 0.001). Furthermore, the two internal angles deviated substantially from the intended 90°, measuring 98° and 85°, respectively. This geometric inaccuracy resulted in non-parallel alignment of the opposing bone interfaces. The procedure was completed in 5 min and 31 s. Furthermore, examination of the osteotomy margins revealed cracks and edge carbonization, as well as elevation of blackened periosteum. Reflecting the macroscopic irregularity, the oscillating saw group displayed a higher surface roughness, with an S_q_ value of 78.51 μm.

### 3.3. Multi-Scale Morphological and Micro-Structural Analysis

#### 3.3.1. Observation Results Under Microscopes

Group L: After laser ablation, the osteotomy surfaces were smooth, and the original microstructure of the bone was retained. Multi-modal microscopy (light microscope, SEM, LSCM) confirmed the preservation of intact cortical and trabecular architecture in Group L, with open intertrabecular spaces and no residual debris.

**Figure 3:**
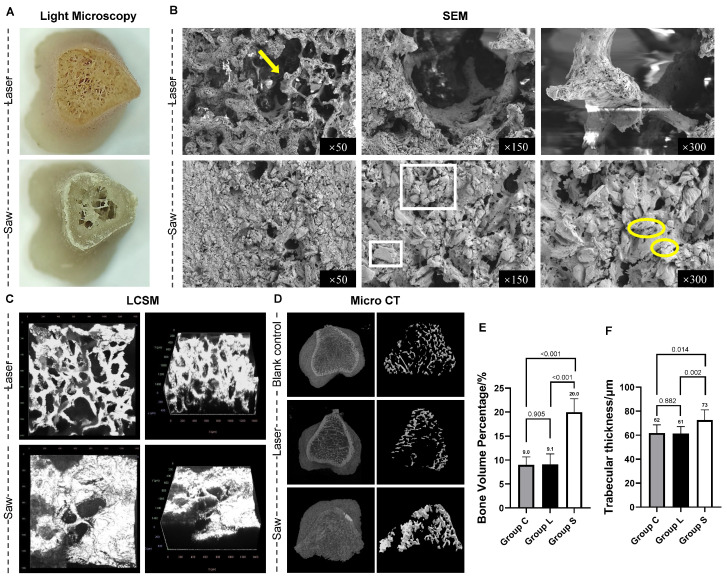
Multi-modal imaging and quantitative microarchitectural analysis of osteotomy surfaces of the rat tibia specimens. (**A**) Macroscopic view of the osteotomy surfaces under a light microscope. (**B**) Representative scanning electron microscopy (SEM) images of the osteotomy surfaces in Group L (laser) and Group S (saw), showing clear structures of trabecular bone (yellow arrow) can be seen in the laser osteotomy samples. A large amount of bone debris (white squares) and microcracks (yellow circles) could be seen in the mechanical osteotomy group. (**C**) Three-dimensional reconstruction of the bone-trabecular interface from laser scanning confocal microscopy (LSCM) in Group L and Group S. Orthogonal LSCM views reveal a sharp interface in Group L, in contrast to the obstructive debris layer in Group S that completely obscures discernible trabecular structure across the entire 600 µm scanned depth. (**D**) Representative three-dimensional micro-computed tomography (micro-CT) reconstructions of the osteotomy site, demonstrating cortical and trabecular architecture. It can be seen that the trabecular bone structure in Group L was clear, and its morphology was similar to that of the blank control group. (**E**,**F**) Quantitative analysis of bone volume fraction (BV/TV) and trabecular thickness (Tb.Th) in micro-CT.

Group S: The osteotomy surfaces were severely damaged, and normal trabecular bone morphology was hardly recognizable. Microcracks in bone trabeculae caused by mechanical vibration damage can also be observed. Plenty of bone debris piled up and filled the trabecular spacing. LSCM examination revealed that the bone debris formed a confluent, obstructive layer that completely obscured the visualization of trabecular details to a depth of 600 µm. As shown in Figure 3A–C and Table 2.

#### 3.3.2. Observation Results Under Micro-CT

The typical images of Micro-CT scanning results are shown in Figure 3D.

Blank control (intact rat tibia): Revealing the original morphology and structure of bone tissue.

Group L: The laser-treated surfaces were characterized by sharp, well-defined margins. Within the cortical bone, clearly patent vascular canals were preserved without signs of thermal melting or occlusion, while the underlying trabecular network remained structurally intact.

Group S: The mechanical saw resulted in an irregular, serrated surface profile with significant cortical fragmentation. This mechanical trauma extended into the cancellous region, causing severe trabecular disruption and the substantial accumulation of radio-opaque bone debris that obscured the original micro-architecture.

The L group exhibited BV/TV of 9.1 ± 2.2% and Tb.Th of 61 ± 6 μm, which showed no significant differences compared with the blank control group (9.0 ± 1.7% and 62 ± 7 μm, all *p* > 0.05), but was greatly lower than those in the S group (20.0 ± 2.8% and 73 ± 9 μm, all *p* < 0.001), as shown in Table 2.

### 3.4. Results of Histological Analysis

**Figure 4:**
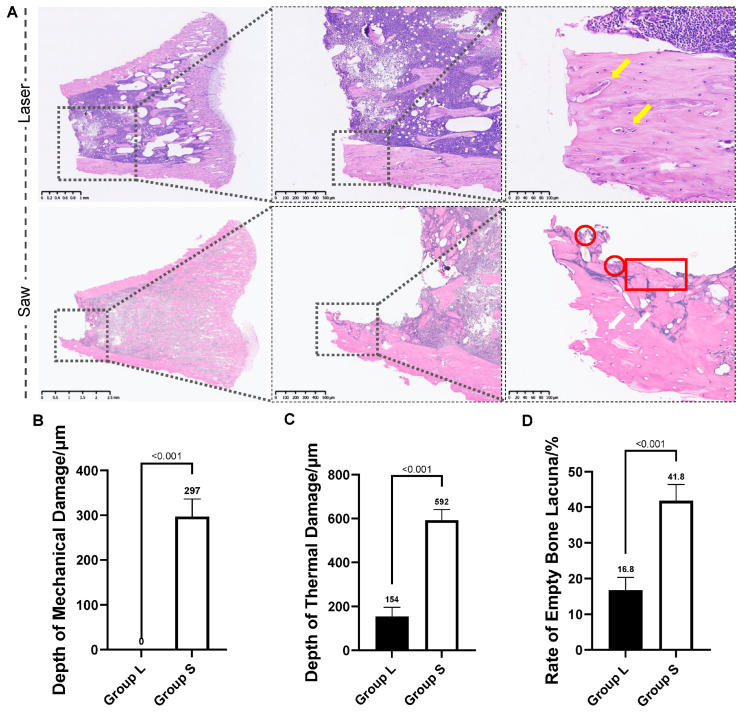
Comparison of histological characterization and quantitative analysis of bone tissue damage between Er:YAG laser ablation and oscillating saw osteotomy. (**A**) Representative H&E-stained images of the osteotomy surfaces. The bone surfaces after laser osteotomy were smooth and neat, and the osteocytes and vascular canals (yellow arrow) beneath the osteotomy margin were in good shape. While in Group S, after mechanical osteotomy, obvious thermal necrosis margins could be seen, including thermal necrosis cell clusters (red circle), accumulation of cortical bone debris (red square), and a large number of empty bone lacunas (white arrow), forming after bone cell necrosis. (**B**–**D**) Quantitative assessment of osteotomy-induced damages: (**B**) thermal damage depth, (**C**) mechanical damage depth (notably, no measurable mechanical damage zone was observed in the Group L), and (**D**) empty osteocyte lacunae rate.

Group L: Following laser ablation, the osteotomy margin was smooth with no discernible zone of thermal necrosis in the cortical bone. Critically, the majority of osteocyte lacunae adjacent to the cut edge retained intact nuclei. The underlying vascular canals and, similarly, the trabecular architecture in the medullary cavity remained structurally normal (see Figure 4A).

Group S: Histological examination of the mechanically osteotomized bone revealed an irregular cutting edge with evident distortion and fragmentation of the bone matrix. Focal areas showed hypereosinophilic condensation of the matrix. In the adjacent region, osteocytes within lacunae exhibited either pyknosis or complete absence, resulting in empty lacunae. Furthermore, aggregates of thermally necrotic cells were observed directly beneath the osteotomy site (see Figure 4A).

The Group L showed a thermal damage depth of 154 ± 42 μm, and an empty lacunae rate of 16.8 ± 3.6%. Both parameters were significantly lower than those in the Group S (592 ± 49 μm and 41.8 ± 4.5%, all *p* < 0.001). A mechanical damage zone with a depth of 297 ± 40 μm was present at the osteotomy margin in the Group S (see Figure 4B–D, and Table 2).

### 3.5. Results of Early Cell–Bone Interactions: Survival, Infiltration, and Cytoskeleton Formation of BMSCs

**Figure 5:**
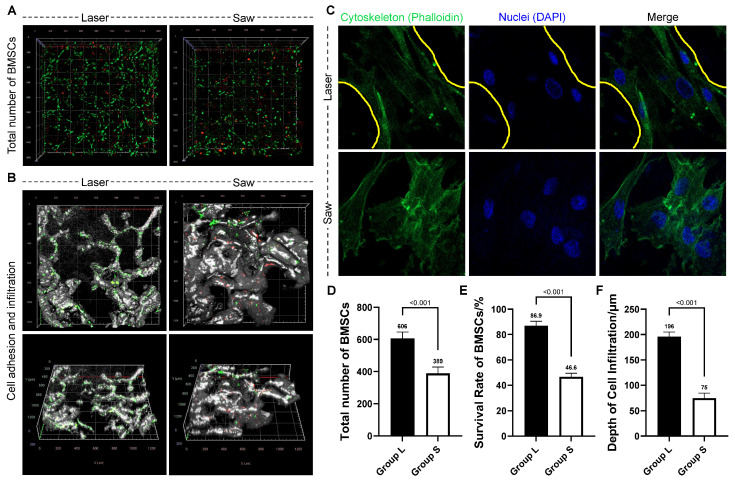
Functional evaluation of bone marrow-derived mesenchymal stem cells (BMSCs) response to the osteotomy interfaces. (**A**) Representative live/dead staining of adherent BMSCs on different osteotomy surfaces (bone microstructure not emphasized), showing more alive BMSCs in Group L. (**B**) Representative live/dead staining of adherent BMSCs on different osteotomy surfaces (showing bone microstructure). In Group L, BMSCs uniformly adhered to the structurally clear bone trabeculae, exhibited active expansion, and infiltrated deep into the bone. On the contrary, in Group S, BMSCs were only distributed in the surface layer with limited expansion due to the damage of the bone trabecular structure. (**C**) The BMSCs cytoskeletal morphology on different osteotomy surfaces (Nuclei: DAPI, blue; Cytoskeleton: Phalloidin, green). It was shown that BMSCs in Group L were arranged orderly and distributed along the bone trabeculae (yellow lines indicate the edges of the bone trabeculae). (**D**–**F**) Quantitative analysis of cell-bone interactions: (**D**) total number of adherent BMSCs, (**E**) survival rate of BMSCs, and (**F**) the infiltration depth of BMSCs into the bone matrix.

The interaction of BMSCs with the osteotomy interface was visualized via LSCM (Figure 5A,B). In Group L, where the 3D trabecular microstructure was preserved, BMSCs exhibited uniform adhesion and active expansion along the trabecular walls, with most cells displaying a characteristic spindle shape and extensive spatial distribution within the porous network. In contrast, on the saw-cut surfaces of Group S, cell distribution was restricted to the superficial layer, with BMSCs forming irregular aggregates around residual trabecular fragments and demonstrating limited expansion. The L group showed a total of 606 ± 40 adherent cells, with a viability rate of 86.9 ± 3.6% and an infiltration depth of 196 ± 9 μm. Corresponding values for the S group were 389 ± 39, 46.6 ± 2.8%, and 75 ± 10 μm, respectively. The differences in various parameters between the two groups were statistically significant (*p* < 0.001), as shown in Table 2.

Cytoskeletal staining (Figure 5C) further delineated the distinct cellular responses between groups. In Group L, BMSCs were evenly dispersed without overlap, aligning along the underlying trabeculae, and exhibited a normal spindle morphology with clearly polarized actin cytoskeletons. In stark contrast, cells in Group S formed irregular aggregates, displayed predominantly elliptical or aberrant shapes with minimal cytoskeletal polarity.

## 4. Discussion

This study aimed to examine the feasibility and biocompatibility of Er:YAG laser osteotomy in complex bone surgery, with particular emphasis on macro-operative performance and early biological response. To this end, a dedicated solid-state Er:YAG laser system was developed, enabling the execution of non-linear osteotomy geometries difficult to achieve with conventional mechanical instruments. Complex Z-shaped and curved osteotomies were first performed on fresh ovine bone to assess geometric controllability and operational feasibility. Subsequently, a multi-level biological evaluation—including surface morphology, histological assessment, and cellular behavior analysis—was conducted using an SD rat bone model to interrogate microstructural preservation and initial cytocompatibility.

The ovine bone model, with its well-established anatomical and biomechanical similarities to human bone [27,28], provided a robust platform for evaluating the macro-operational capabilities of laser osteotomy. Comparative analysis revealed that laser-assisted Z osteotomy (modeling the clinical Scarf osteotomy for hallux valgus correction [29] and femoral lengthening procedures [30]) achieved a precise and consistent gap of 1.14 ± 0.05 mm, in stark contrast to the wider and highly variable gap produced by the oscillating saw (2.70 ± 0.41 mm, range: 2.2–3.3 mm, *p* < 0.001).

From a clinical standpoint, this high-precision gap offers more than just a geometric advantage; it facilitates tighter cortical apposition and enhances inter-fragmentary stability. Our quantitative roughness analysis further confirmed that laser-ablated surfaces were significantly smoother than the jagged interfaces created by mechanical saws. In orthopedic surgery, minimizing the osteotomy gap is a prerequisite for promoting primary contact healing and reducing the reliance on extensive callus formation, which in turn accelerates functional recovery [31]. While the current preliminary study did not directly quantify biomechanical strength or long-term bone formation volumes, the precision and biological favorability of the laser interface suggest a clear potential for improved inter-fragmentary stability. Future clinical validation should ideally employ Micro-CT imaging to quantify bone bridging and torsional loading tests to confirm whether these interfacial advantages translate into superior macro-scale mechanical integrity during the bone remodeling phase.

A critical observation highlights the inherent imprecision of mechanical sawing: the average saw gap was more than twice the physical thickness of the blade itself (1.0 mm). This discrepancy does not stem from the blade dimension, but rather from unavoidable blade deflection, operator variability, and microfracture propagation—factors that collectively lead to uncontrolled bone loss and elevated surface roughness. The precision of laser osteotomy, therefore, derives from its ability to circumvent these very sources of error [32]. This superiority in precision stems from the fundamental non-contact ablation mechanism [33], which intrinsically avoids the surface defects—such as burrs and micro-cracks, and macroscopic irregularities—that are inevitably introduced by mechanical sawing [34]. In contrast, the laser’s cut gap closely matched the minimal thickness of the saw blade itself. This demonstrates that laser-mediated material removal is confined to the photothermal focal volume, thus eliminating collateral mechanical widening while producing a flatter, more uniform cutting plane. These defects, acting alongside increased roughness as stress concentrators, compromise initial mechanical stability and are known to detrimentally affect the biological process of bone healing [35]. By confining material removal to the photothermal focal volume, laser technology ensures that the resulting interface is both geometrically optimized for stable fixation and biologically prepared for rapid integration.

Furthermore, laser technology provides a geometric freedom unattainable with conventional instruments [36]. The successful execution of an arc-shaped osteotomy (gap: 1.16 ± 0.08 mm) in this study serves as direct proof of concept. This approach holds immediate transformative potential for procedures like high tibial osteotomy (HTO). Conventional HTO, constrained by instrument limitations, typically employs straight or wedge-shaped cuts that inevitably remove bone, creating a structural defect and elevating the risks of non-union and refracture [37]. In contrast, the continuous, defect-free osteotomy plane enabled by laser ablation not only maximizes native bone preservation and eliminates the inherent healing risks associated with defects but also facilitates unparalleled intraoperative controllability for fine, continuous adjustment of lower limb alignment [38].

Taken together, the principal advancement of laser osteotomy at the macro-operative level lies in the geometric versatility and sub-millimeter precision conferred by its non-contact mechanism. This technological attribute allows it to overcome the limitations of traditional instruments in anatomically constrained and morphologically complex regions such as the pelvis and spine, providing a pivotal tool for advancing truly precision orthopedic surgery [39].

Based on the aforementioned macroscopic advantages, we hypothesized that laser osteotomy would better preserve bone microstructure. To test this, we performed an in-depth analysis using an SD rat bone model, which is well-established for investigating early cellular responses at the bone-healing interface [40,41].

Our findings confirm that laser osteotomy achieves exceptional preservation of the native bone architecture at the microscopic scale. Multi-modal imaging revealed that, compared to the mechanically osteotomized surfaces—which were severely damaged, riddled with micro-cracks, and clogged with debris—the laser-ablated surfaces were smooth and the trabecular network remained intact. Laser scanning confocal microscopy (LSCM) further quantified this disparity, demonstrating that mechanical damage extended more than 600 µm into the bone, whereas collateral damage from laser ablation was strictly confined. This structural preservation carries direct biological significance: the intact trabeculae provide a physical scaffold for subsequent repair [42], while the substantial amount of impacted debris generated by mechanical osteotomy can activate osteoclasts [43] and trigger a sustained inflammatory response [44], thereby disrupting the homeostatic microenvironment essential for bone healing [45].

Quantitative micro-CT analysis revealed a critical finding: although the bone volume fraction (BV/TV: 20.0 ± 2.8% vs. 9.1 ± 2.2%) and trabecular thickness (Tb.Th: 73 ± 9 μm vs. 61 ± 6 μm) were significantly higher in the mechanical group, this apparent increase represents a measurement artifact resulting from the compaction of high-density debris into the marrow cavities [46]. Clinically, such impacted debris may form avascular necrotic zones, provoking excessive inflammatory responses and increasing the risk of postoperative infection [43]. In contrast, the parameters of the laser group showed no statistically significant difference from those of the blank control group (BV/TV: 9.0 ± 1.7%; Tb.Th: 62 ± 7 μm), providing definitive evidence for the high-fidelity preservation of bone microstructure. Consequently, at the microscopic level, laser technology—by virtue of its non-contact nature and spatially confined energy delivery—successfully circumvents the deep structural disruption and debris-induced adverse biological reactions inherent to mechanical osteotomy [47].

Histological analysis with H&E staining provided direct evidence at the cellular level: the laser group exhibited a significantly reduced thermal damage depth (154 ± 42 μm vs. 592 ± 49 μm) and empty lacunae rate (16.8 ± 3.6% vs. 41.8 ± 4.5%). This profound cytoprotection is attributed to the fundamental mechanism of Er:YAG laser ablation. Its 2940 nm wavelength is intensely absorbed by water in the bone matrix, leading to rapid superficial vaporization (ablation) with minimal conductive thermal diffusion [48]. Consequently, a high proportion of osteocytes at the interface remain viable—a cellular reservoir critical for orchestrating early bone remodeling [49]. In contrast, the oscillating saw induces coagulation necrosis through prolonged frictional heating and uncontrolled thermal conduction [50]. Furthermore, the non-contact nature of laser ablation completely obviated the direct mechanical damage zone (297 ± 40 μm thick in the saw group) caused by blade pressure. This zone of matrix compaction and fragmentation not only compromises the initial stability of the bone fragments but also generates a field of debris that physically impedes biological integration by obstructing vascular ingrowth and osteoprogenitor cell migration [51]. Therefore, laser osteotomy uniquely mitigates the dual iatrogenic injuries—thermal and mechanical—that are inherent to conventional sawing. By preserving both the structural integrity and cellular vitality at the critical healing interface, it establishes a pro-regenerative microenvironment conducive to more predictable and robust bone repair [52].

Superior preservation of bone microstructure by laser osteotomy elicits a more favorable biological response in host cells. Functional validation via in vitro cell culture assays confirmed this finding: BMSCs cultured on laser-ablated surfaces exhibited markedly higher total cell counts (606 ± 40 vs. 389 ± 39), cell viability rates (86.9 ± 3.6%vs. 46.6 ± 2.8%), and infiltration depths (196 ± 9 µm vs. 75 ± 10 µm). Notably, these cells retained a typical spindle-shaped morphology with clear cytoskeletal polarization. This enhanced cellular activity is primarily attributed to the unique surface roughness and preserved microtopography of laser-ablated bone interfaces. In contrast to oscillating saws, which induce mechanical compaction of bone tissue, Er:YAG laser ablation preserves the native three-dimensional trabecular architecture of bone. While laser-ablated surfaces display greater microscale roughness than mechanically prepared ones, this topographical feature acts as a bioactive substrate: it substantially increases the effective surface area for cell–matrix interactions and provides critical anchoring sites for focal adhesions, which are indispensable for initiating cell–matrix signaling and sustaining cellular colonization on microstructured bone surfaces [53,54]. By comparison, oscillating saws generate a smear layer—a biologically inert interface composed of compacted bone debris and denatured proteins that occludes the underlying vascular canals [55]. This forms a pseudosmooth yet biocompatibly unfavorable microenvironment, where BMSCs aggregate and exhibit abnormal morphologies due to the absence of stable attachment sites. Despite its uniform appearance, the low inherent roughness of the smear layer lacks essential topographical cues to facilitate deep cellular infiltration. The significant reduction in cell viability and limited cellular expansion observed in Group S are likely driven by two key factors: physical obstruction by the smear layer and exposure to proinflammatory cytokines released from mechanically damaged bone tissue [56,57]. Collectively, these findings suggest that the laser-ablated interface, characterized by its functional micro-topography, provides a bio-favorable environment that may facilitate early cell-bone interactions conducive to the subsequent regenerative process.

A major limitation of this study is the substantially longer operative time of laser osteotomy versus conventional mechanical sawing. To address this translational barrier, future technical optimizations should prioritize increasing laser repetition rates and adopting advanced scanning strategies (e.g., parallel or spiral paths). Integrating robotic assistance or surgical navigation could further enable continuous, high-speed motion control—critical for maximizing efficiency while retaining sub-millimeter precision. Clinically, translation to operating theatres demands strict compliance with safety protocols, including high-efficiency smoke evacuation for surgical plume management and wavelength-specific protective eyewear. Practical challenges persist, however, such as the need for unobstructed line-of-sight and equipment footprint constraints in the sterile field, which require ergonomic, fiber-guided delivery systems for resolution. Biologically, our evaluations were confined to ex vivo and short-term in vivo models, focusing primarily on early cell-bone interactions. A key technical limitation is the lack of quantitative nano-scale surface topography mapping (e.g., via Atomic Force Microscopy) to complement our SEM observations—data that would deepen insights into the fractal dimensions of the ablated surface. Though 72 h assays confirm enhanced biocompatibility, the underpinning molecular mechanisms remain incompletely elucidated. Critical to resolving this gap is the need for molecular kinetic assays (e.g., Ki-67 or EdU staining) to decouple proliferation rates from initial adhesion efficiency. To validate the hypothesized role of focal adhesions in sensing laser-modified topography, future studies should integrate immunofluorescence staining for vinculin or paxillin, paired with Western blot analysis of integrin-FAK signaling pathways. Such molecular-level evidence would definitively link the preserved 3D microtopography to the observed superior cellular colonization. Finally, the absence of long-term validation for angiogenesis and key osteogenic pathways (e.g., BMP or Wnt), coupled with inherent differences in bone remodeling rates between rats and humans, warrants caution when extrapolating these findings to weight-bearing orthopaedic clinical sites.

In summary, this study demonstrates that Er:YAG laser osteotomy concurrently achieves high-precision geometric engineering and exceptional preservation of the bone tissue microenvironment. It transforms bone cutting from a contact-dependent, mechanically traumatic procedure into a non-contact, energy-controlled photonic process. This fundamental advance positions it as a key enabling technology for advancing personalized and precision orthopedic surgery.

## 5. Conclusions

In summary, this study provides a foundational proof-of-concept for Er:YAG laser-assisted osteotomy, demonstrating its potential for achieving high-precision geometric cutting and superior preservation of the bone microenvironment. Our findings suggest that the non-contact, water-confined ablation mechanism mitigates the thermal and mechanical damage inherent to conventional saws, thereby creating a pro-regenerative interface that supports early stem cell adhesion, viability, and infiltration. While a significant trade-off between operative efficiency and cutting precision currently exists, this work establishes a mechanistic basis for the technology’s clinical potential. Future research focused on optimizing laser parameters, implementing surgical safety protocols (e.g., plume management and eye protection), and conducting long-term assessments in load-bearing skeletal sites will be essential. Consequently, while this study should be viewed as an early-stage mechanistic evaluation rather than a final clinical protocol, it contributes foundational data toward the potential advancement of precision surgical techniques.

## Figures and Tables

**Table 1:** Parameters of Er:YAG Laser system.

Parameters	Value
Wavelength	2940 nm
Frequency	10 Hz
Pulse Width	400 μs
Scanning speed	10 mm/s
Single pulse energy	1.2 J
Cooling mode	Water spray

**Table 2:** The comparison between results of osteotomy with the Er:YAG laser and the oscillating saw.

Results		Group L *	Group S **	*p* Value
Marco-scale evaluation of osteotomy in ovine femurs	Duration of Z-shape Osteotomy	23 m 15 s	5 m 31 s	-
	Gap of Z-shape Osteotomy/mm	1.14 ± 0.05	2.70 ± 0.41	<0.001
	Surface Roughness (Sq)/μm	15.79	78.51	-
Mirco-scale evaluation of osteotomy in rat tibias	Bone Volume Percentage/%	9.1 ± 2.2	20.0 ± 2.8	<0.001
	Trabecular thickness/μm	61 ± 6	73 ± 9	0.002
	Depth of mechanical Damage/μm	0	297 ± 40	<0.001
	Depth of Thermal Damage/μm	154 ± 42	592 ± 49	<0.001
	Rate of Empty Bone Lacuna/%	16.8 ± 3.6	41.8 ± 4.5	<0.001
	Total number of BMSCs	606 ± 40	389 ± 39	<0.001
	Survival Rate of BMSCs/%	86.9 ± 3.6	46.6 ± 2.8	<0.001
	Depth of Cell Infiltration/μm	196 ± 9	75 ± 10	<0.001

* Group L: osteotomy with the Er:YAG laser. ** Group S: osteotomy with the oscillating saw.

## Data Availability

The original data presented in the study are included in the article; further inquiries can be directed to the corresponding authors.

## Supplementary Materials

The following supporting information can be downloaded at https://www.mdpi.com/article/10.3390/bioengineering13020237/s1, Figure S1: light microscope; Figure S2: SEM-laser(50×); Figure S3: SEM-laser(150×); Figure S4: SEM-laser(300×); Figure S5: SEM-saw(50×); Figure S6: SEM-saw(150×); Figure S7: SEM-saw(300×); Figure S8: LSCM-laser; Figure S9: LSCM-saw; Figure S10: Micro CT-blank control; Figure S11: Micro CT- laser; Figure S12: Micro CT-saw; Figure S13: he staining-laser; Figure S14: he staining-saw; Figure S15: BMSCs-laser; Figure S16: BMSCs-saw; Figure S17: BMSCs infiltration-laser; Figure S18: BMSCs infiltration-saw; Figure S19: BMSCs cytoskeleton(Phalloidin)-laser; Figure S20: BMSCs Nuclei (DAPI)-laser; Figure S21: BMSCs merge-laser; Figure S22: BMSCs cytoskeleton(Phalloidin)-saw; Figure S23: BMSCs Nuclei (DAPI)-saw; Figure S24: BMSCs merge-saw; Figure S25: Illustration of the Volume of Interest (VOI) for Micro-CT analysis; Table S1: Reliability analysis of the parameter measurement

## Abbreviations

The following abbreviations are used in this manuscript:

BMSCs	bone marrow-derived mesenchymal stem cells
BV/TV	bone volume/total volume
DAPI	4′,6-diamidino-2-phenylindole
EDTA	ethylenediamine tetraacetic acid
Er:YAG	Erbium-doped Yttrium-Aluminum-Garnet
HE	Hematoxylin and Eosin
HTO	high tibial osteotomy
LSCM	saw scanning confocal microscope
PBS	phosphate-buffered saline
PFA	paraformaldehyde
SD rats	Sprague-Dawley rats
SEM	scanning electron microscopy
Tb.Th	trabecular thickness
VOI	volume of interest
