# Supplementary material for: A Multiscale Evaluation of Erbium-Doped Yttrium-Aluminum-Garnet Laser Osteotomy: Integrating Macroscopic and Cellular Analyses
Source: Bioengineering (Basel). 2026 Feb 18;13(2):237. doi: 10.3390/bioengineering13020237 (PMC12938275; doi:10.3390/bioengineering13020237)
Supplement: Supplementary file 1 [file bioengineering-13-00237-s001.zip › Fig.S25-caption.pdf]

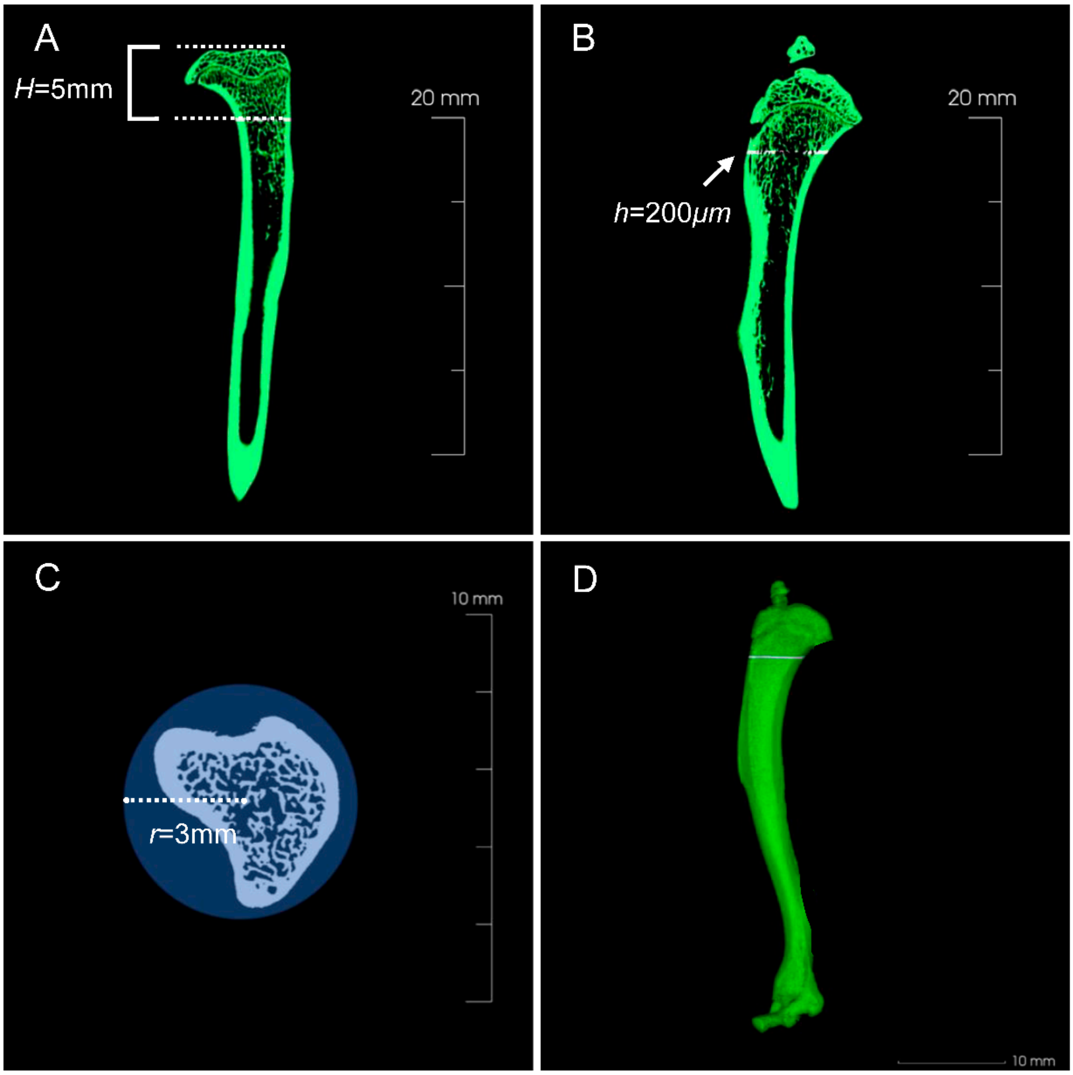

Supplementary figure 25. Illustration of the Volume of Interest (VOI) for Micro-CT analysis. (A) Coronal view of the proximal tibia indicating the osteotomy plane located 5mm distal to the articular surface. (B) Representative slice highlighting the specific region of interest (h = 200μm). (C) Transverse (axial) view demonstrating the circular ROI with a radius (r) of 3mm centered on the bone shaft. (D) 3D reconstructed model of the proximal tibia.
