# Supplementary material for: A Multiscale Evaluation of Erbium-Doped Yttrium-Aluminum-Garnet Laser Osteotomy: Integrating Macroscopic and Cellular Analyses
Source: Bioengineering (Basel). 2026 Feb 18;13(2):237. doi: 10.3390/bioengineering13020237 (PMC12938275; doi:10.3390/bioengineering13020237)
Supplement: Supplementary file 1 [file bioengineering-13-00237-s001.zip › Supplementary Table S1.pdf]

Supplementary table S1 Reliability analysis of the parameter measurement.

	**ICC (interobserver)***	**95% Confidence Interval**	
		Lower bound	Upper bound
Gap of Z-shape Osteotomy/mm	0.84	0.78	0.89
Depth of Mechanical Damage/μm	0.93	0.88	0.96
Depth of Thermal Damage/μm	0.85	0.76	0.91
Rate of Empty Bone Lacuna/%	0.90	0.81	0.98
Total number of BMSCs	0.91	0.86	0.94
Survival Rate of BMSCs/%	0.86	0.81	0.98
Depth of Cell Infiltration/μm	0.83	0.76	0.90

* ICC, intraclass correlation efficient.
