# Supplementary figures and images for: A Multiscale Evaluation of Erbium-Doped Yttrium-Aluminum-Garnet Laser Osteotomy: Integrating Macroscopic and Cellular Analyses
Source: Bioengineering (Basel). 2026 Feb 18;13(2):237. doi: 10.3390/bioengineering13020237 (PMC12938275; doi:10.3390/bioengineering13020237)

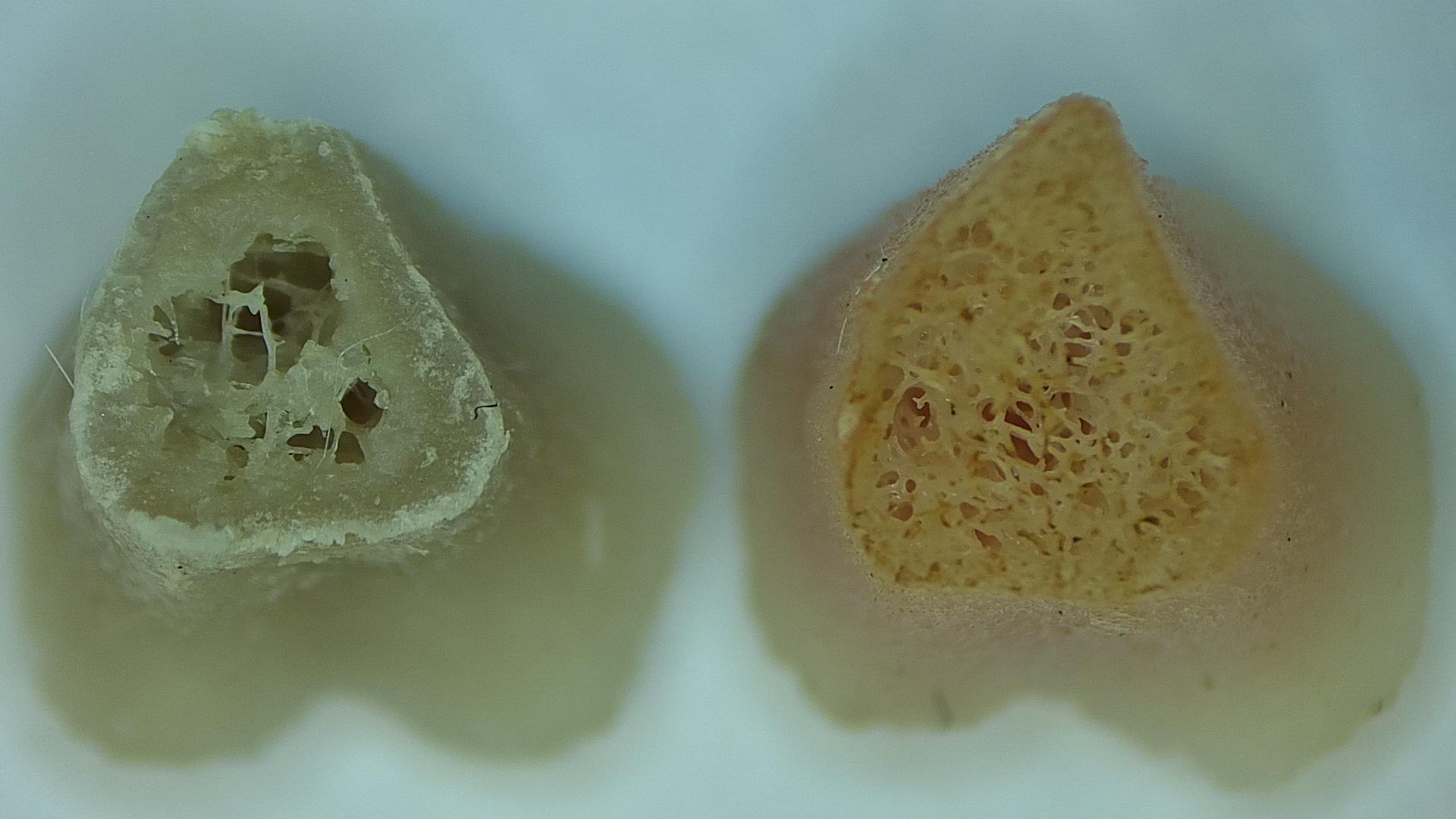

Supplement: Supplementary file 1 [file bioengineering-13-00237-s001.zip › Fig.S1 light microscope.jpg]

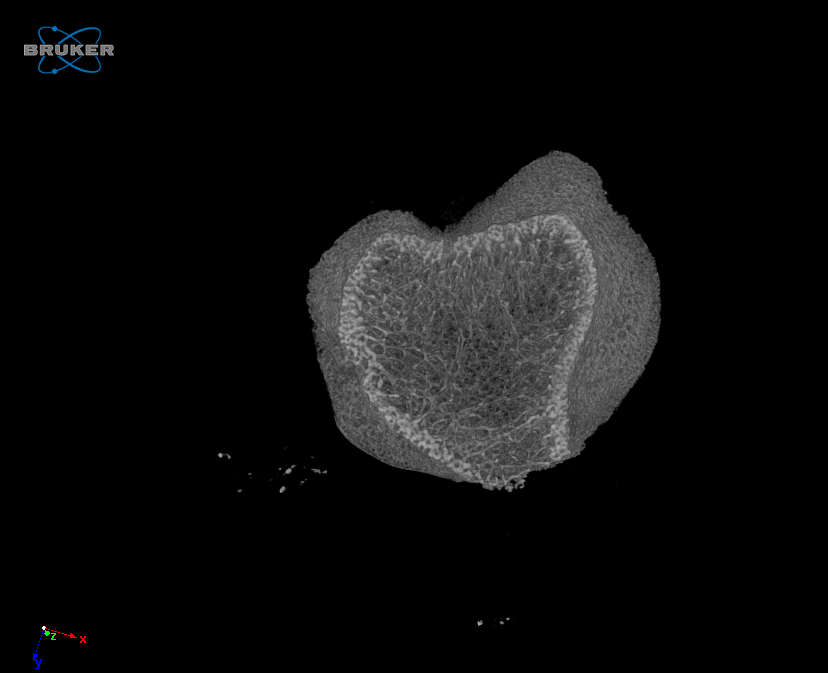

Supplement: Supplementary file 1 [file bioengineering-13-00237-s001.zip › Fig.S10 MicroCT-blank control.bmp]

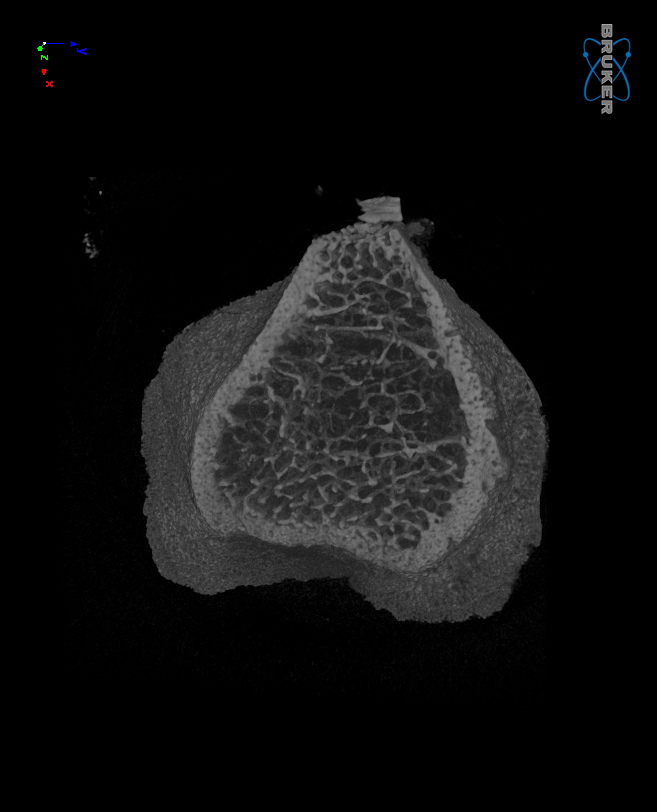

Supplement: Supplementary file 1 [file bioengineering-13-00237-s001.zip › Fig.S11 MicroCT-laser.bmp]

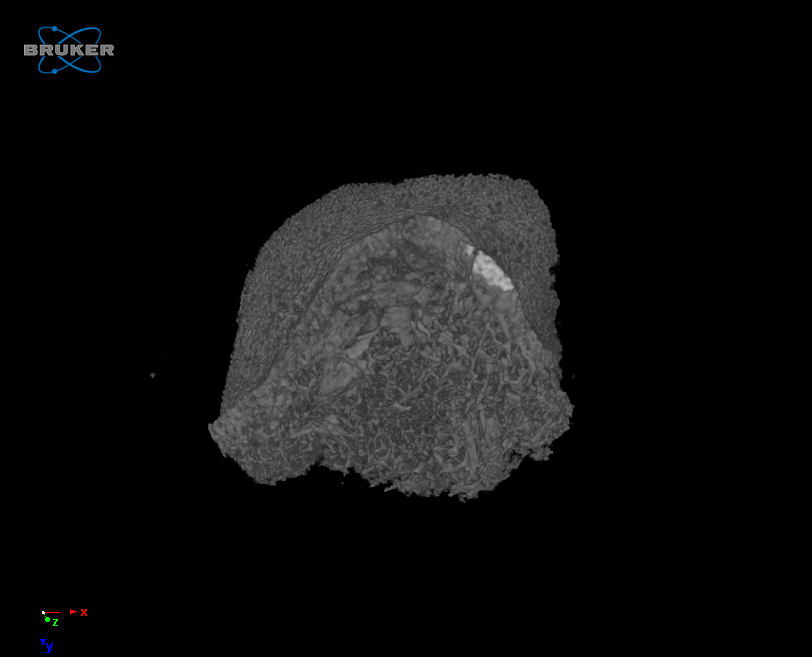

Supplement: Supplementary file 1 [file bioengineering-13-00237-s001.zip › Fig.S12 MicroCT-saw.bmp]

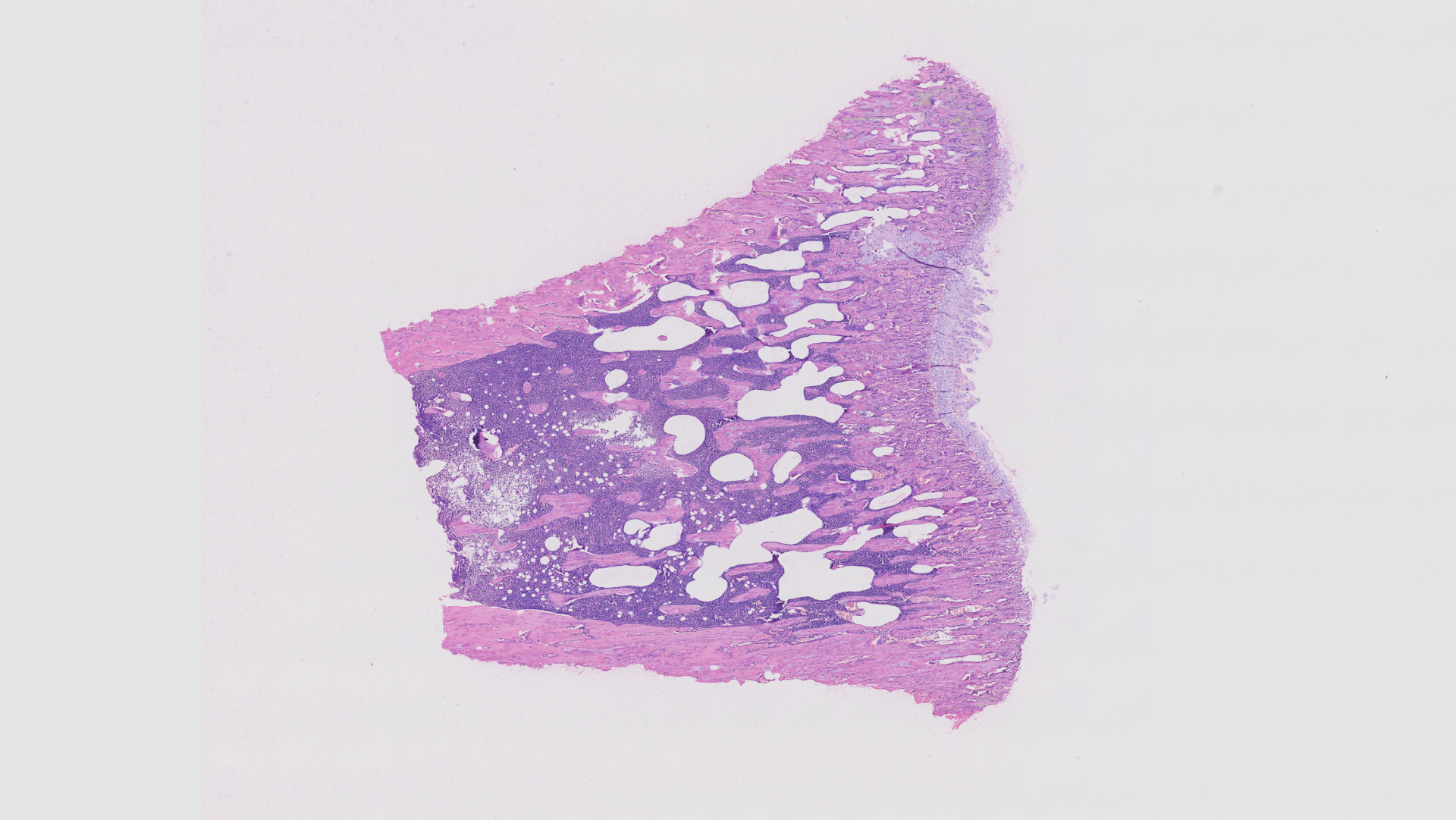

Supplement: Supplementary file 1 [file bioengineering-13-00237-s001.zip › Fig.S13 he staining-laser.tif]

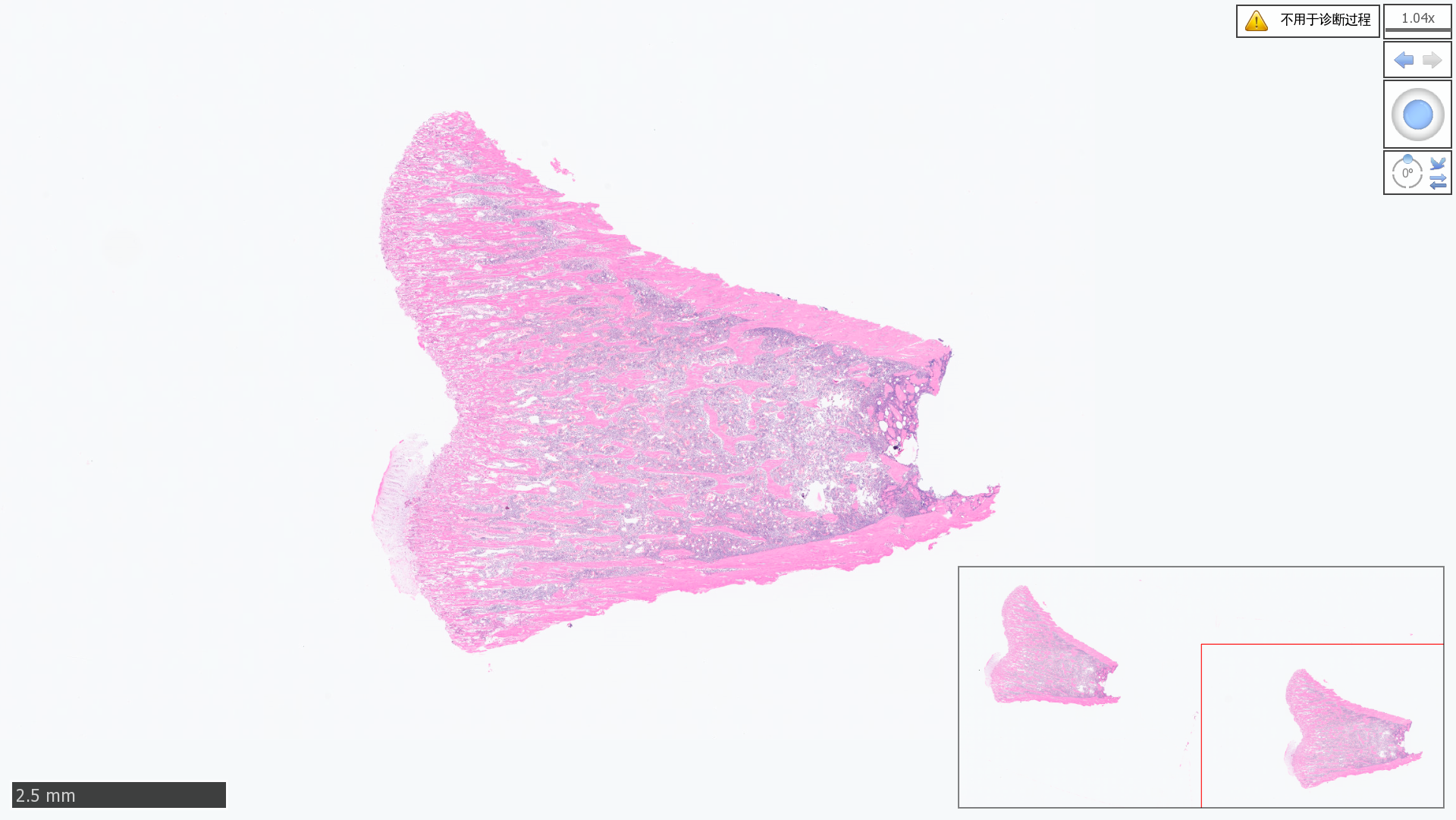

Supplement: Supplementary file 1 [file bioengineering-13-00237-s001.zip › Fig.S14 he staining-saw.tif]

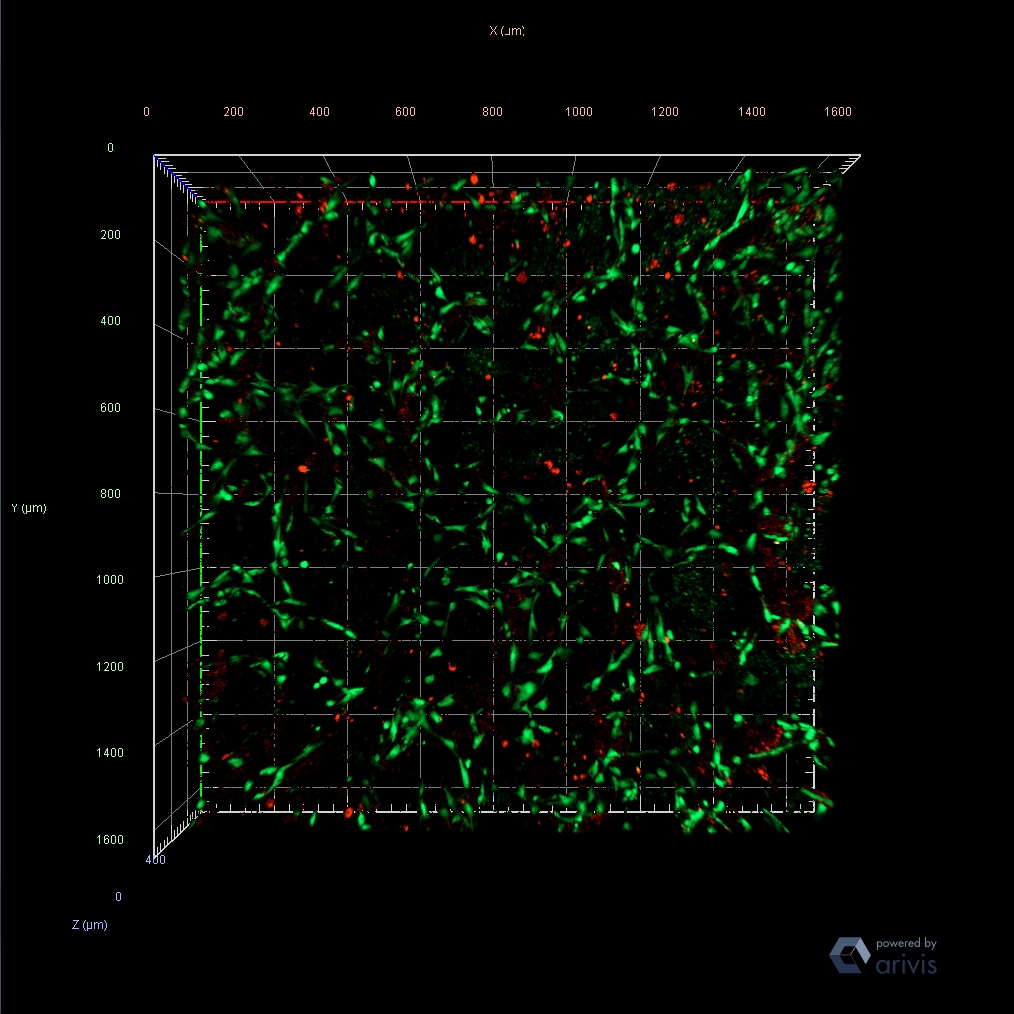

Supplement: Supplementary file 1 [file bioengineering-13-00237-s001.zip › Fig.S15 BMSCs-laser .png]

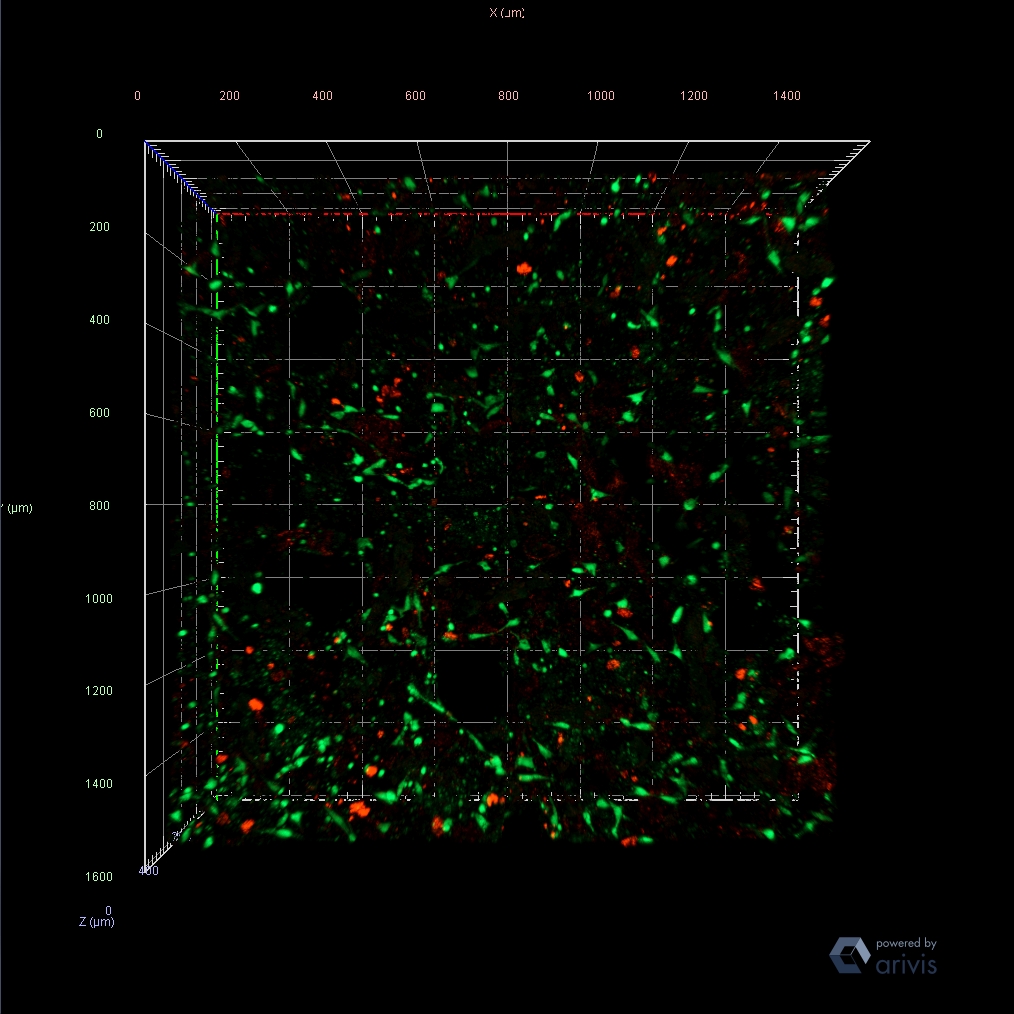

Supplement: Supplementary file 1 [file bioengineering-13-00237-s001.zip › Fig.S16s BMSCs-saw.png]

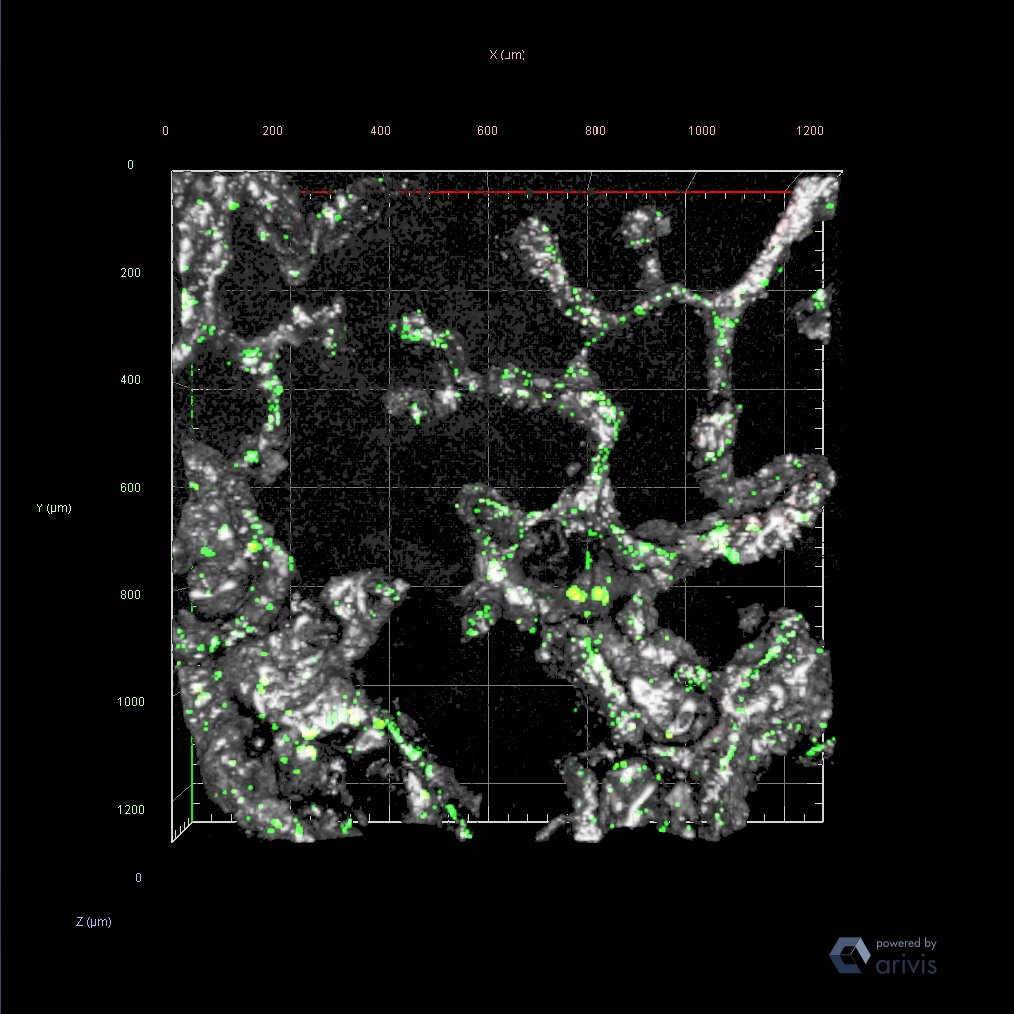

Supplement: Supplementary file 1 [file bioengineering-13-00237-s001.zip › Fig.S17 BMSCs infiltration-laser.png]

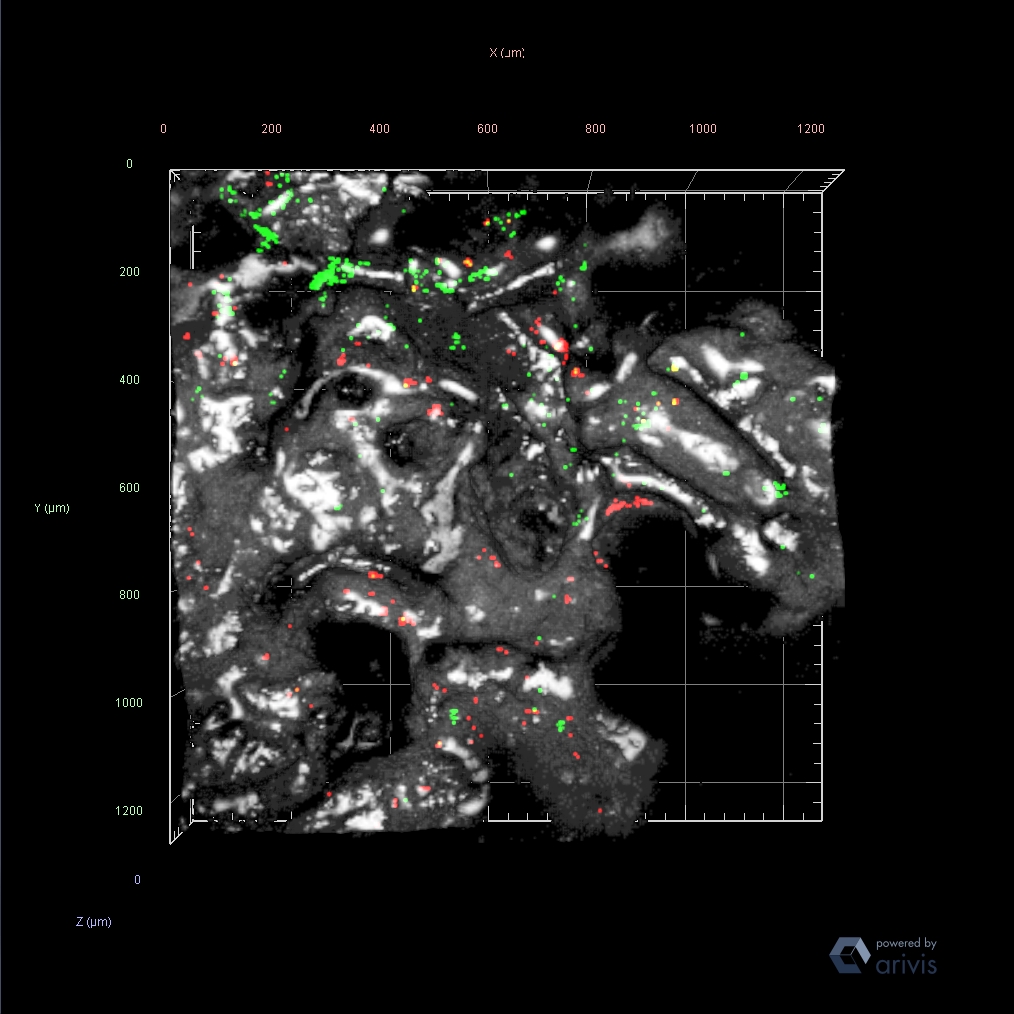

Supplement: Supplementary file 1 [file bioengineering-13-00237-s001.zip › Fig.S18 BMSCs infiltration-saw.png]

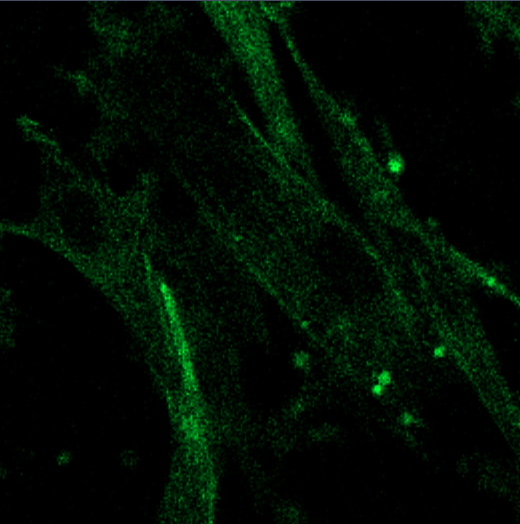

Supplement: Supplementary file 1 [file bioengineering-13-00237-s001.zip › Fig.S19 BMSCs cytoskeleton(Phalloidin)-laser.png]

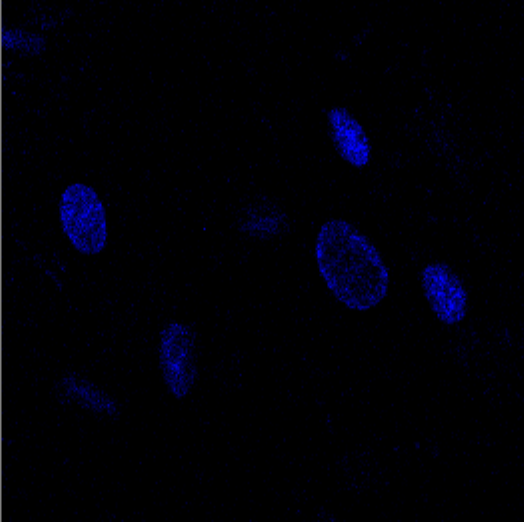

Supplement: Supplementary file 1 [file bioengineering-13-00237-s001.zip › Fig.S20 BMSCs Nuclei (DAPI)-laser.png]

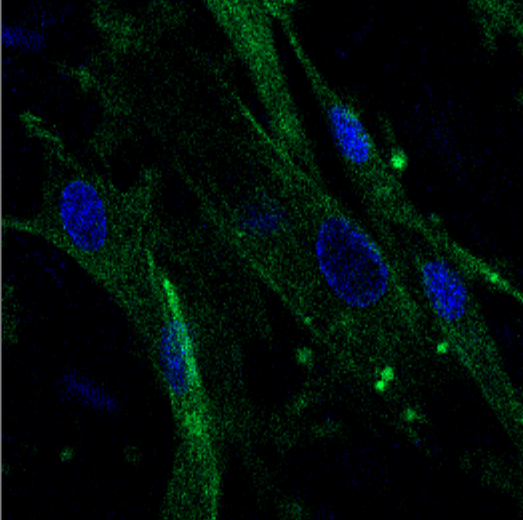

Supplement: Supplementary file 1 [file bioengineering-13-00237-s001.zip › Fig.S21 BMSCs merge-laser.png]

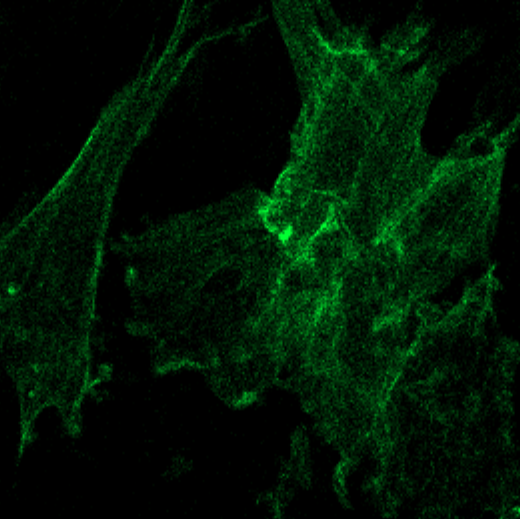

Supplement: Supplementary file 1 [file bioengineering-13-00237-s001.zip › Fig.S22 BMSCs cytoskeleton(Phalloidin)-saw.png]

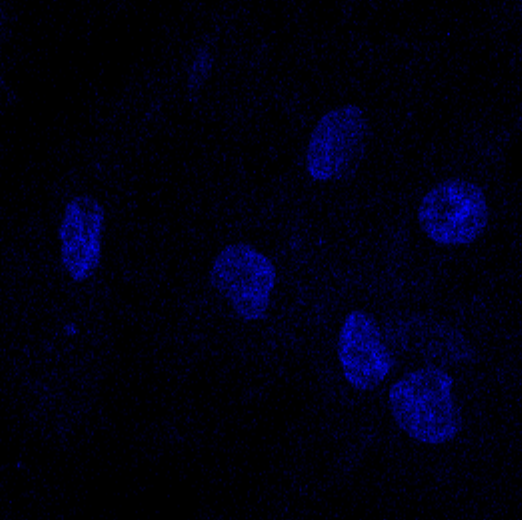

Supplement: Supplementary file 1 [file bioengineering-13-00237-s001.zip › Fig.S23 BMSCs Nuclei (DAPI)-saw.png]

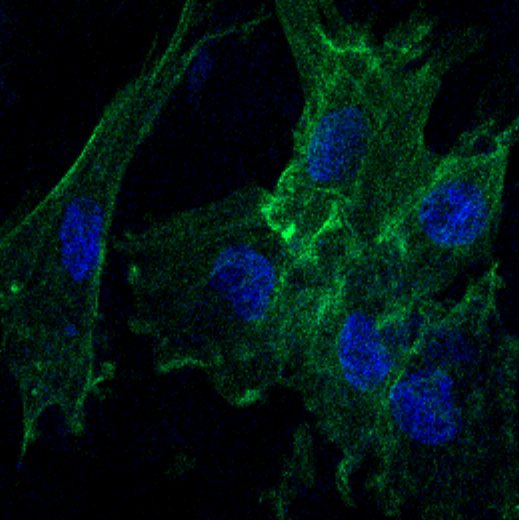

Supplement: Supplementary file 1 [file bioengineering-13-00237-s001.zip › Fig.S24 BMSCs merge-saw.png]

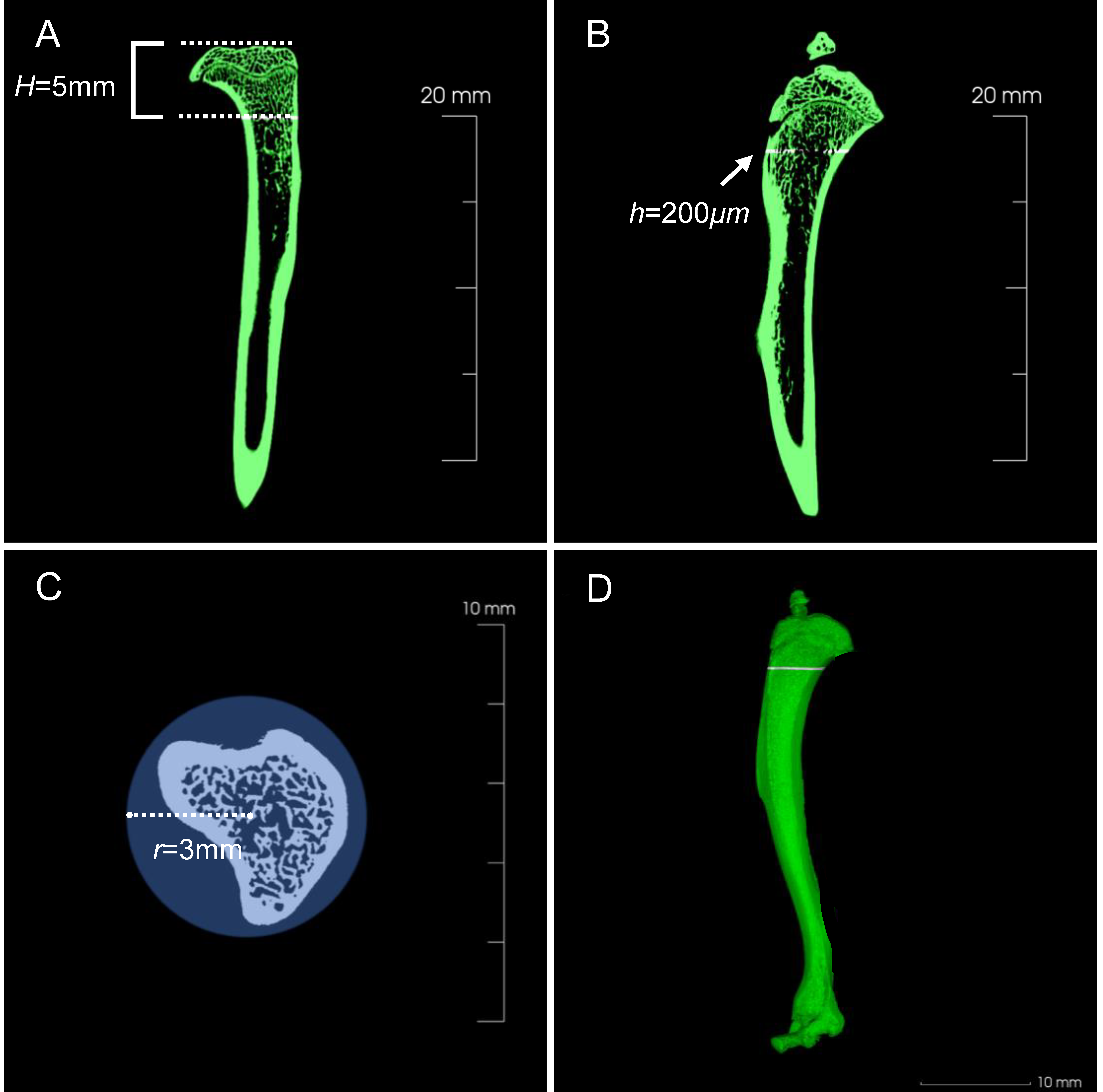

Supplement: Supplementary file 1 [file bioengineering-13-00237-s001.zip › Fig.S25 Illustration of the Volume of Interest (VOI) for Micro-CT analysis..tif]

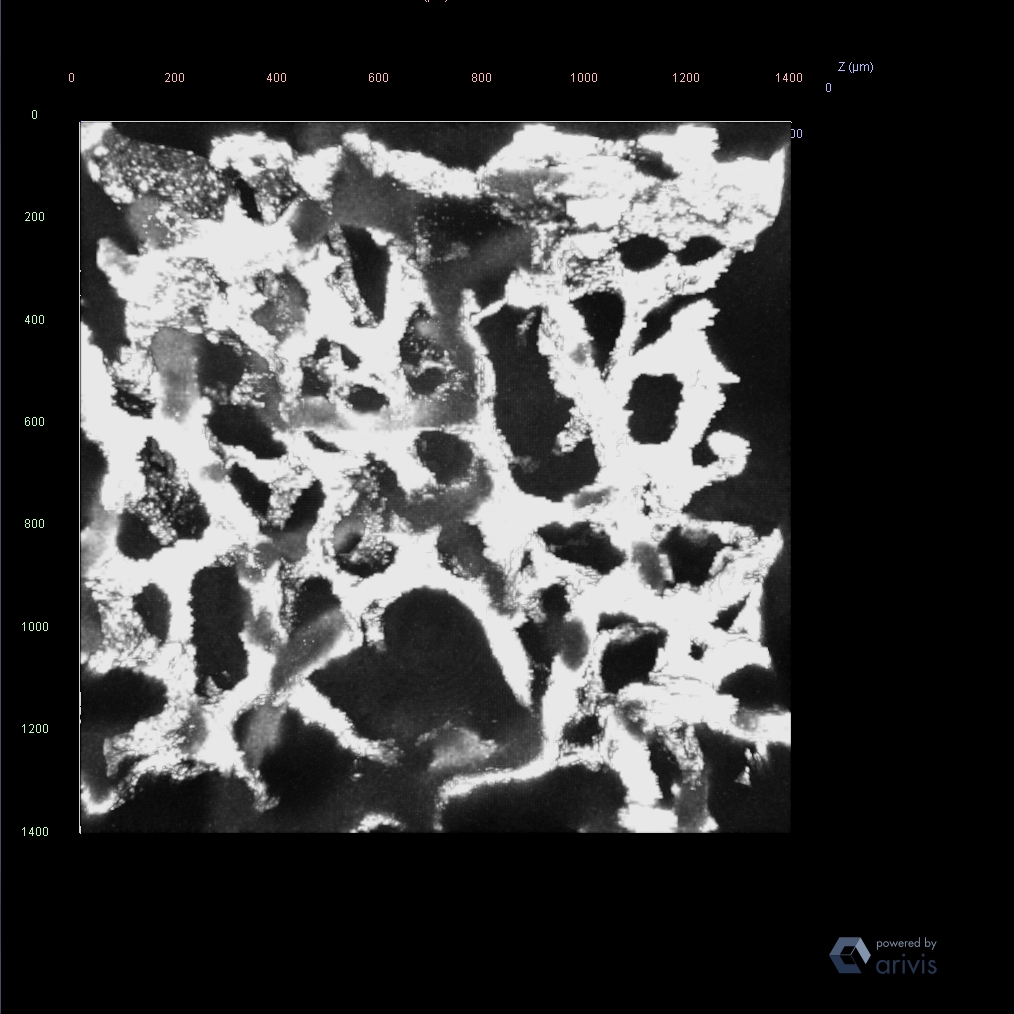

Supplement: Supplementary file 1 [file bioengineering-13-00237-s001.zip › Fig.S8 LSCM-laser.png]

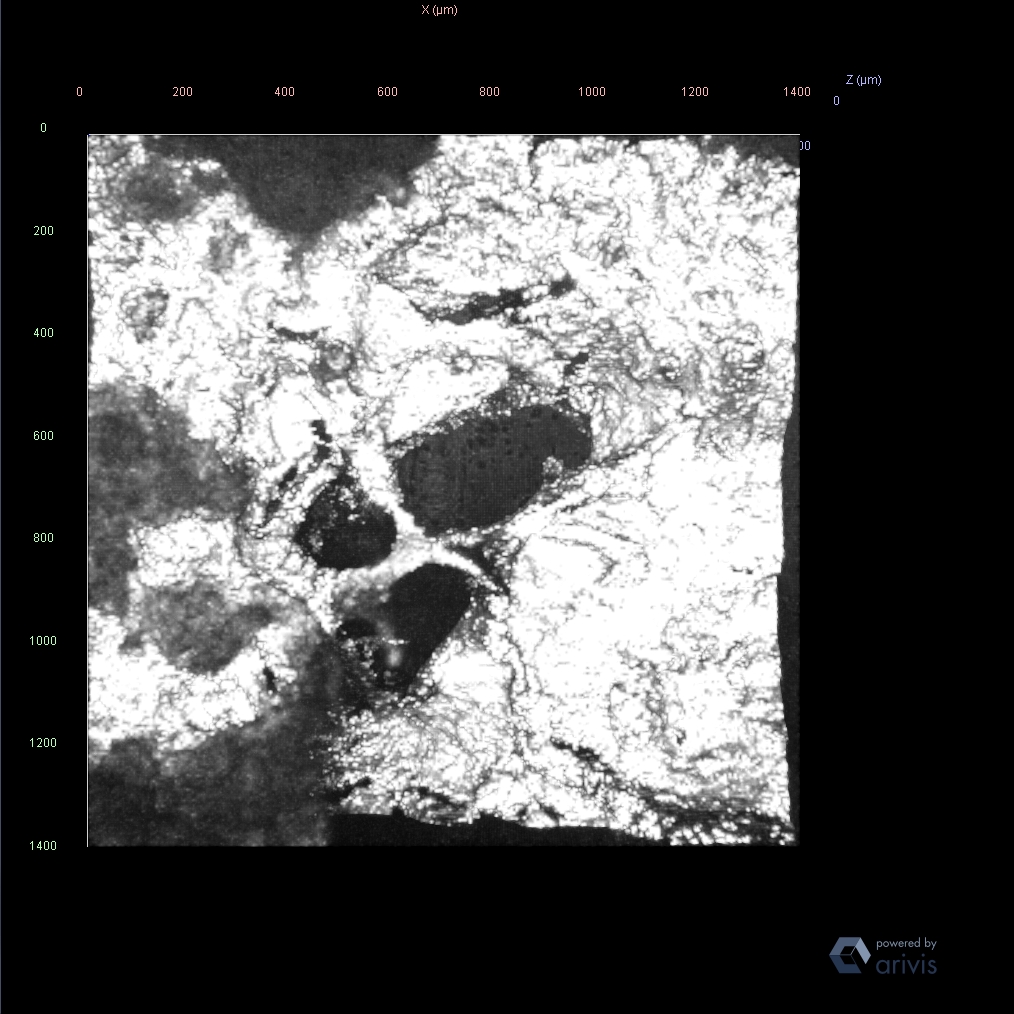

Supplement: Supplementary file 1 [file bioengineering-13-00237-s001.zip › Fig.S9 LSCM-saw.png]
